# A hybrid unsupervised—Deep learning tandem for electrooculography time series analysis

**DOI:** 10.1371/journal.pone.0236401

**Published:** 2020-07-21

**Authors:** Ruxandra Stoean, Catalin Stoean, Roberto Becerra-García, Rodolfo García-Bermúdez, Miguel Atencia, Francisco García-Lagos, Luis Velázquez-Pérez, Gonzalo Joya

**Affiliations:** 1 University of Craiova, Craiova, Romania; 2 Universidad de Málaga, Málaga, Spain; 3 Universidad Técnica de Manabí, Portoviejo, Ecuador; 4 Cuban Academy of Sciences, La Habana, Cuba; 5 Center for Research and Rehabilitation of Hereditary Ataxias, Holguín, Cuba; Korea National University of Transportation, KOREA, REPUBLIC OF

## Abstract

Medical data are often tricky to get mined for patterns even by the generally demonstrated successful modern methodologies of deep learning. This paper puts forward such a medical classification task, where patient registers of two of the categories are sometimes hard to be distinguished because of samples showing characteristics of both labels in turn in several repetitions of the screening procedure. To this end, the current research appoints a pre-processing clustering step (through self-organizing maps) to group the data based on shape similarity and relabel it accordingly. Subsequently, a deep learning approach (a tandem of convolutional and long short-term memory networks) performs the training classification phase on the ‘cleaned’ samples. The dual methodology was applied for the computational diagnosis of electrooculography tests within spino-cerebral ataxia of type 2. The accuracy obtained for the discrimination into three classes was of 78.24%. The improvement that this duo brings over the deep learner alone does not stem from significantly higher accuracy results when the performance is considered for all classes. The major finding of this combination is that half of the presymptomatic cases were correctly found, in opposition to the single deep model, where this category was sacrificed by the learner in favor of a good accuracy overall. A high accuracy in general is desirable for any medical task, however the correct identification of cases before the symptoms become evident is more important.

## 1 Introduction

Machine learning, and recently deep learning (DL), are truly becoming virtual medical assistants to clinicians by improving medical diagnosis and allowing more physician—patient time, despite the still open issues of lack of transparency and susceptibility to adversarial examples [1]. More pragmatically, in October 2019, the software Vara based on artificial intelligence was approved by the German regulatory body for automatic breast cancer screening [2]. The software filters the images of the healthy class and thus enables the radiologists to focus on the problematic mammograms.

Therefore, the scientific literature abounds in successful DL models constructed for different data types and areas of medicine (as summarized in 2). Nevertheless, while DL models are becoming more accurate by the day, the raw data coming from medicine is yet not as straightforward to analyze and to get fitted with a good model. An example of such a medical scenario where a direct time series deep model does not work immediately is provided in the current paper for a bio-signal data collection. For this task, it is no longer the case of a single signal connected to a ground truth label, as in the usual signal applications, but a group of several signals from tests at given intervals, sometimes with different shapes, that finally get the same label. Hence a special pre-processing step must be appointed prior to the actual DL training.

We thus target the complex computational diagnosis of spino-cerebral ataxia of type 2 from electrooculography time series. This is a neurodegenerative hereditary disorder that cannot be cured, yet impeded to progress with a timely detection. There are no noticeable clinical symptoms for an early diagnosis of a presymptomatic condition and only expensive genetic testing can confirm it. An alternative cheaper manner for screening is represented by the electrooculographic examination of a patient. The patient follows an object appearing suddenly in different parts of the screen and the machine records the weak electrical potentials that are triggered by the eye movement. In a simplified description, this movement during the examination time represents a saccade and its behavior is further analyzed by the physician. The test is repeated a number of times, especially if there are reasons to suspect a presymptomatic form from the past saccades. As a consequence, a register of saccades will be created for every single patient.

The complexity of this medical problem stems from two sides. The first is that a presymptomatic saccade is only slightly different in shape in comparison to a healthy one. The second issue appears because all the saccades in a patient register are eventually labeled with the same class found from a (not specified) number of tests, irrespective of the fact that in many more others the movement appears healthy or, on the contrary, presymptomatic. Both aspects pose a serious problem for machine learning methodologies that strive to discover the relationship between shape and diagnosis class. As such, their accuracy of discrimination cannot go beyond 66% [3, 4], no matter the techniques employed.

The present study therefore proposes a solution to this classification task through a combined approach of supervised—unsupervised learning. Based on an initial natural and positionally-related grouping discovered by the clustering method of Self-Organizing Maps (SOM), the training saccades get relabeled in a (weighted) way that is linked more to the similarity of shapes than to some unknown and biased (by other unavailable medical information) decision. The relabeled training set is then fed to a DL method and the resulting model will eventually provide the prediction regarding the diagnosis class of the test registers.

The paper has the following structure. Recent studies for applying DL approaches to data from the medical field are referred in 2. The data set is described in section 3. The hybrid approach is outlined in section 4, where the relabeling procedure is shown in subsection 4.1 and the constructed DL architecture is presented in subsection 4.2. The experiments, given in section 5, have a twofold aim: on the one hand, they examine the parameters that control the case relabeling, mainly in the problematic case of telling apart healthy vs. presymptomatic conditions; on the other hand, they envisage the setup for the application of the DL. The conclusions enunciate the findings in the closing section 6.

## 2 Related works

Medicine is one of the most rewarding fields of modeling for DL. Its success stemmed principally from the existence of its inner mechanisms, which allow automatic feature extraction and hence require no further implication of the physician at the training level, as in traditional machine learning techniques in the field (see e.g. [5, 6] for recent reviews). While most of DL applications in medicine are primarily directed towards images from different procedures and devices, signal processing is also a prominent area of DL interest.

On the image interpretation side, histopathological samples are one frequent topic of DL papers [7]. In this context, ensemble methods have been proposed in order to assist the pathologist in cancer grading, by providing an additional degree of confidence that highlights samples deserving further study [8]. Image segmentation by machine learning methods, and its relation to human experts’ annotation is another remarkable direction [9]. Breast cancer multi-classification on the public BreakHis data set has been tackled by appointing an approach that performs semantic hierarchical feature learning followed by a convolutional neural network (CNN) [10]. Another recent study on the micro-environment of the breast cancer tissue using DL allows for cancer grading from digital analysis of images that provide quantitative metrics of disease [11]. A survey of other DL approaches to cancer diagnosis from pathology images is provided by [12]. While many of these works focus on breast cancer, colorectal cancer slides have also been quite frequently analyzed by CNN, in order to obtain survival predictions [13], or to grade cancer staging [14]. On a different note, images from radiology (MR and CT) have also been analyzed by DL methodologies, e.g. for detection of significant anatomical features [15]. A discussion on the success of these methods in the presence of small data samples, class imbalance and lack of explainability are addressed in [16], whereas recent reviews of the interplay between DL algorithms and radiology are given in [17, 18]. Finally, a recent subject of application for CNN architectures is the analysis of ophthalmology images, e.g. for prediction of prognosis in diabetic retinopathy [19] or macular edema classification [20].

Several attempts have been made to transfer the success of DL techniques in visual processing to the context of biomedical signals [21], where time series data from medicine are usually connected to cardiology, i.e. electrocardiograms (ECG). An ensemble of echo state networks uses ECG data for the classification of arrhythmias [22], as for this type of data a recurrent architecture is appropriate. A standard NN is appointed in [23] for the detection of ischemia from ECG. A convolutional U-net is applied to ECG recordings in [24], being able to deal with variable length data, which is a common problem of temporal signals, in contrast to images. Some research has also been dedicated to the analysis of electroencephalograms (EEG) by DL methods, e.g. for the diagnosis of dementia [25] or seizure prediction [26]. As in the case of ECG, recurrent neural networks are suitable for dealing with the temporal features of EEG, and they have found diagnostic applications in the prognosis of epilepsy [27]. The Long-Short Term Memory (LSTM) is a specialized recurrent neural network that has been applied to lapse detection from EEG processing [28]. Assisted mobility devices have been designed based upon the analysis of electrooculography data [29]. In general, biomedical signal data are limited and imbalanced [21], thus further research effort is required to overcome these limitations.

The problem we tackle in this study stems from the need to assess neurological disorders, by using electrooculography data in time series format. It will be modelled by a CNN-LSTM tandem, which was the model that best captured the information from the data, as revealed by experimentation. Moreover, the particularities of the data also require a pre-processing unsupervised step.

## 3 Materials: The saccadic data set

The data set used in this paper was built from electrooculographic signals, extracting the segments where saccades ocurred. These signals are also known as electrooculograms (EOG) and are measures of the electrical potential between the cornea and the eye membrane, representing the angular position of the eyes. The signals were recorded using the Otoscreen electronystagmographer from Jaeger-Toennies with a sampling frequency of 200 Hz. The data collection comes from the Centro para la Investigación y Rehabilitación de las Ataxias Hereditarias (CIRAH), Holguín, Cuba.

To build the data set, a total of 85 EOG records from different subjects were used. Each of the records is from a subject whose diagnosis falls into one of the following 3 categories: healthy or control (denoted further by C), presymptomatic (P) and ataxia present or sick (S). Each one of these records contains a set of tests in which saccadic visual stimuli of 4 different angles (10°, 20°, 30°, 60°) were used. The saccadic segments are extracted from the horizontal signals of these tests using a simple KMeans based segmentation algorithm. The full distribution of the data set is detailed in Table 1.

**Table 1:** Data set distribution by registers, saccades, angles of the visual stimuli and diagnosis class.

	Registers	S10	S20	S30	S60	Total Saccades
**Control**	38	537	865	807	695	2904
**Presymptomatic**	18	266	441	403	388	1498
**Sick**	29	365	469	469	248	1551
**Total**	85	1168	1775	1679	1331	5953

**Fig 1:**
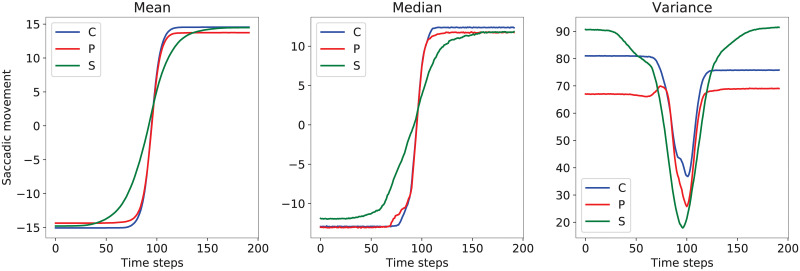
Means, medians, and variance for the saccades (units: Degrees) in the training set, for each class in turn, with respect to time (units: Milliseconds).

**Fig 2:**
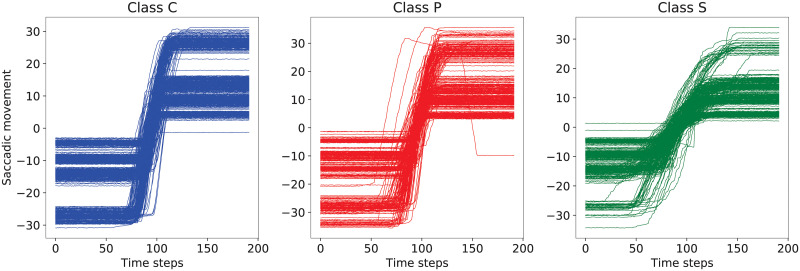
Examples of saccades (units: Degrees) labeled as C, P and S, respectively, taken from the test set, with respect to time (units: Milliseconds).

Finally, a window with a length of 192 samples is taken from the center of each saccade in their horizontal position signal, resulting in a matrix of shape (5953, 192). Then, this matrix is scaled by the angle of the stimuli to obtain saccades with around the same amplitude (30°). Also, to normalize the direction of the saccades, right-to-left ones are swapped vertically. Fig 1 illustrates the mean, median and variance plots for each class in turn for the training set. An image of the appearance of saccades for each type of diagnosis outcome is provided in Fig 2. The plots in both figures indicate a higher resemblance between the saccades of type C and those of type P.

The present form of the data set was recompiled in February 2019 by CIRAH by changing the label to healthy for 11 registers initially mislabeled as presymptomatic. The change was triggered by the examination of an external parameter, i.e. the longitude of mutation, for these cases.

The data set used in this work can be downloaded for research purposes from the following link: https://dx.doi.org/10.6084/m9.figshare.11926812.

## 4 Methods: The clustering—Deep learning tandem

A preliminary study in [4] outlined the separate findings of a trio of techniques for both supervised and unsupervised analysis of the previous version of the saccadic data. A temporal CNN from the classification side, and K-means and SOM from the clustering perspective, each discovered different patterns in the shapes of the saccades.

**Fig 3:**
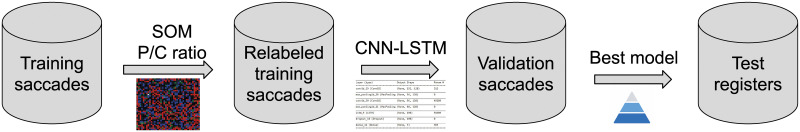
Overview of the proposed approach. Training saccades are relabeled via SOM in combination with the tuning of the P/C (presymptomatic/control) ratio, then the CNN-LSTM is trained on this data, the best model on the validation set is applied on the test saccades, and finally the test registers get classified.

Based on these earlier distinct findings, the aim of the current paper now becomes to hybridize the DL supervised learning with the unsupervised grouping power of the SOM approach. The SOM clustering algorithm was chosen because its found association of saccades was closer to their real arrangement. The 1D CNN is now used for feature extraction and followed by a LSTM component to achieve an enhanced temporal memory of the data. The SOM will ‘correct’ the labels of the training data and the CNN-LSTM will subsequently model these new samples. A threshold to ‘guess’ the physicians’ inclination towards a presymptomatic or control decision, when saccades of both appearances are present, is also included in the new approach. A depiction of the methodological flow is given in Fig 3.

### 4.1 Clustering for training data relabeling

The SOM method [30] is a network that projects an *n*-dimensional data space (pattern space) into a two-dimensional plane (network space), while preserving the topology of the former. This allows the visualization (in two dimensions) of the classification of patterns from a space of *n* dimensions. The neurons in the network have a neighboring relationship to each other in the network space (similar to a fishing net) and each of them has an *n*-dimensional weight vector associated. SOM training assumes that a ‘winning neuron’ is selected for each input pattern, based on the distance from its weight vector to the presented input pattern in the *n*-dimensional space. The weight vectors both of the winning neuron and of those of its neighbors in the network space change in order to be more similar to the corresponding sample in the *n*-dimensional space. At the end of the iterative process, two neighboring neurons in the network space have also adjacent weight vectors in the pattern space. This implies that two data samples that are very similar to each other activate the same winning neuron (or two neighboring neurons in the network space) and that two neighboring neurons in the two-dimensional plane are activated as winning neurons for data samples that are alike.

Thus SOM can be seen as an algorithm for unsupervised grouping or *clustering* of data, where prototypes or cluster representatives appear on the two-dimensional grid that becomes a feature space. This constraint is kept during training, thus it allows for an informative and comprehensible visualization of the resulting grouping. Also, in our particular problem, the clustering results lead to a straightforward relabeling of saccades based on the classes of those positioned on the same point on the map.

**Fig 4:**
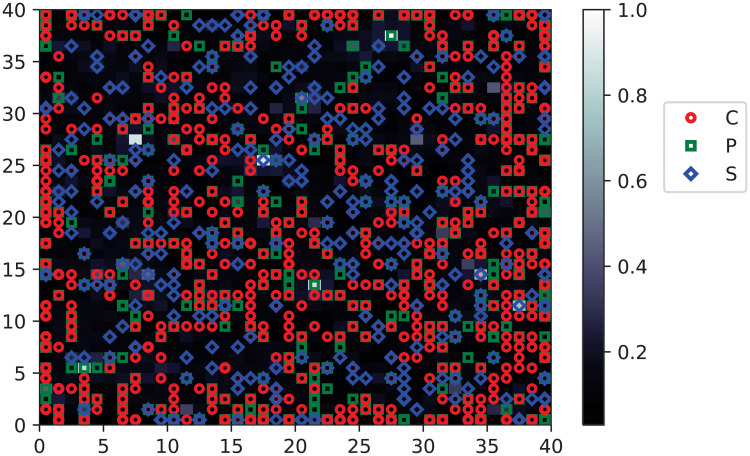
An obtained SOM map on training data. The C samples are in red, the P in green and the S in blue. The overlapping of samples of distinct classes in many points of the map is observable.

At first, the SOM finds the natural grouping of the training saccades. The obtained map contains the localization of every sample based on its shape and independent of the class. An example of a reached SOM map in one of the cross-validation runs can be seen in Fig 4. The figure brings yet another (visual) argument in favor of relabeling a number of saccades, since the red circles (C) and green squares (P) are more than often superimposed on a same position from the map.

The obtained SOM map then reassigns a class for each training sample based on the labels of the saccades mapped at the same position. This occurs because the SOM assigned the saccades to their location based on their similarity, hence the majority class for each position decides the actual label for that point of the SOM map. However, if there is a mixture of healthy and presymptomatic saccades in a certain map position, the establishment of the class corresponding to that location must somehow imitate the expert decision in the screening of a patient. More precisely, a majority label of sick clearly points to a sick tag, whereas otherwise it is the ratio between healthy and presymptomatic counts that tilts the balance in favor of one or the other. This threshold will be selected based on the validation set so as to maintain a good balance between the sensitivity of detecting both presymptomatic and control samples.

### 4.2 Deep learning for the classification of regrouped saccades

While LSTM have been constructed to possess a long-short term memory in order to handle temporal data [31], 1D CNN have recently also proved to be able to model time series within real-world applications either alone [32] or in combination with the former [33]. Therefore, the architecture used herein will be another combination of the two deep networks with 1D CNN being in charge of extracting the features and the LSTM of modeling the time dependencies. Apart from the theoretical precedent, the reason for this tandem stemmed from the significantly poorer results observed during pre-experimentation, when either a LSTM or a CNN was employed alone.

LSTM are deep networks that were built to capture long-term dependencies in the problem data. They do so through a chain of repeating modules, each composed of a cell state and three gate functions interacting with it: the forget gate, the input gate and the output gate. These allow for the temporal information to flow, while some of it gets forgotten and other gets stored.

CNN, on the other hand, has reached popularity due to its success for image analysis, which admit a natural 2D representation. It has been however transposed to other types of problems, one of which being temporal data. In this case, a 1D input is accepted and the inherent convolutions between kernels and data are performed in a number of layers.

Once the training saccades are relabeled following the SOM grouping, the DL CNN-LSTM architecture is appointed to learn the correspondence between shape and ‘corrected’ outcome. The established architectural flow is composed of two convolutional layers and a LSTM one. The model with the best accuracy on the validation set of saccadic samples is taken and applied to the test collection of saccades, and then the prediction is performed at the register level, with the majority label for its saccades giving the class.

### 4.3 The SOM-DL algorithm

The proposed procedure is outlined by Algorithm 1. The SOM clustering technique is applied to the training collection and the obtained map (line 1) gives the ‘cell-mates’ for every training saccade (lines 4-5). Based on the majority label of the mates, the current sample gets another label reassigned. If the majority are sick, then the saccade gets the S label (lines 6-7). Conversely, if there is no presymptomatic item, then the control samples will dominate the current position and set the C label (lines 8-9). Intuitively following the medical procedure, there can be only a bunch of saccades in a register that are already indicative of presymptomatic. Or, contrarily, the appearance of a number of saccades may point towards presymptomatic, but still the patient is considered healthy. Therefore, a threshold will be appointed in the current methodology to find the ratio between the C and P labels that lead the saccade to one of these classes (lines 10-14).

The procedure will search different values for this threshold (lines 2-19). The relabeling of the training saccades is performed based on this parameter and its optimal value if selected based on the CNN-LSTM output on the registers in the validation set. So, once the training saccades are relabelled, the CNN-LSTM architecture is applied on the ‘cleaned’ collection (line 16) and the most balanced model on the validation set, with respect to the sensitivity (recall) for classes C and P, is selected (lines 16-17). The test accuracy is done register-wise, however, at this point, it is the majority label of its inner saccades (predicted by the DL model) that give the final class (lines 20-24).

**Algorithm 1**: The SOM-DL approach: data relabeling by SOM and classification by CNN-LSTM. Recall that a register comprises all the saccades belonging to the same patient.

**Data**: Training, validation and test data set of saccades

**Result**: Predicted label for test registers

1 Create SOM map from training saccades;

2 **for** *manually selected values of threshold* **do**

3  **for** *each training saccade* **do**

4   Find the position of the saccade on the map;

5   Count the number of labels |*C*|, |*P*|, |*S*| in each class for the samples positioned in the same location on the SOM map;

6   **if** *majority label* = *S* **then**

7    Relabel the saccade with S;

8   **else if** |*P*| = 0 **then**

9    Relabel the saccade with C;

10   **else if** |*C*|/|*P*|<*threshold* **then**

11    Relabel with P;

12   **else**

13    Relabel with C;

14   **end**

15  **end**

16  Train CNN-LSTM on the relabeled training saccades;

17  Compute recall for validation registers;

18  Pick CNN-LSTM model corresponding to best recall compromise between C and P;

19 **end**

20 **for** *each test register* **do**

21  **for** *each test saccade* **do**

22   Predict label by CNN-LSTM model;

23  **end**

24  Label register by majority vote of its saccades;

25 **end**

26 Return predicted labels for test registers;

The idea of ‘correcting’ the samples before DL by clustering them first is an inverted notion of the pedagogical approach to information extraction algorithms from black-box models [34]. The latter uses the input—output mapping found by a support vector machine or neural network as a noise remover before extracting rules of decision from the relabeled set. Conversely, the idea in the current study is to use the SOM as an oracle that revises the labels of the saccades according to the grouping it found and the deep neural network to employ the reconsidered class attribution for establishing a model of classification.

## 5 Experimental results

A crucial difficulty for the classifier comes from the fact that all saccades in a register are labeled with its class, although their appearance does not necessarily fit the one of the prototype for that class. This issue appears for the presymptomatic class, where a register contains some saccades that are very similar to the control ones, while having only a small number of saccades with a shape that better characterizes the sick class. The problem is further more complex through the fact that the current data set is unbalanced: there are 38 registers for the control class, 18 for presymptomatic and 29 for the sick. The discrepancy between classes makes the classification task particularly challenging, and reinforces the medical experts’ assessment that the intricate part is to distinguish between classes control and presymptomatic. While the original training set has 1198 saccades of class control, 523 presymptomatic and 524 sick, the changes from applying Algorithm 1 lead to 782 samples for control, 1015 presymptomatic and 448 for sick. At the first glance, the training data appear to become unbalanced by favoring now the presymptomatic class, but the validation samples remain the same and the CNN-LSTM model is selected to be the best performing one on the validation set.

The DL receives an input shape of size (192, 1), following the data shape from section 3. The architecture consists of two consecutive convolutional 1D layers of kernel size 3 and number of filters 128, to which ReLU and max pooling 1D are attached. Following them, a LSTM with 100 units is added, then dropout with a rate of 0.6 is applied, and the final dense layer with a softmax activation with 3 units ends the sequence. This architecture and parametrization was decided from manual pre-experimentation.

The training, validation and test sets are randomly split into an approximate 40%-40%-20% ratio, based on the registers. Precisely, for control there are 15-15-8 registers, for presymptomatic 7-7-4, while for sick these are 12-12-5. The same division is kept for all the experiments in order to avoid favorable splits for some of the tested parameter configurations. For each configuration there are 10 repeated runs performed and the mean results are reported. The CNN-LSTM is trained on the saccades from the training set, validated on the ones that form the registers in the validation set, and the best model found during validation is applied on the test saccades. Finally, the majority class of the saccades from each register in the test set establishes its class.

**Fig 5:**
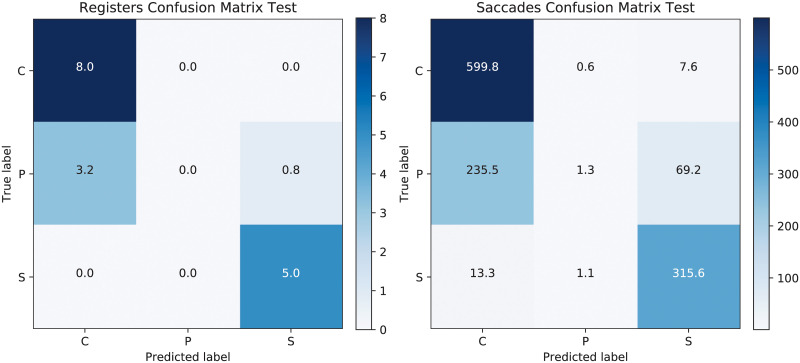
Confusion matrices for the test set when applying CNN-LSTM on the original training data, using registers on the left and saccades on the right. The prediction accuracy for the test registers is 76.47%.

We have carried out an initial experiment by applying the CNN-LSTM directly on the original data set, without any interference from SOM. The results showed that the presymptomatic saccades (and accordingly the registers) are generally mistaken for control ones. Fig 5 illustrates the confusion matrices for the test set for the registers and for the saccades when CNN-LSTM are trained on the unchanged data. Although the control and sick registers are perfectly classified (and almost the same happens for the saccades), there is no register correctly classified for presymptomatic individuals.

**Fig 6:**
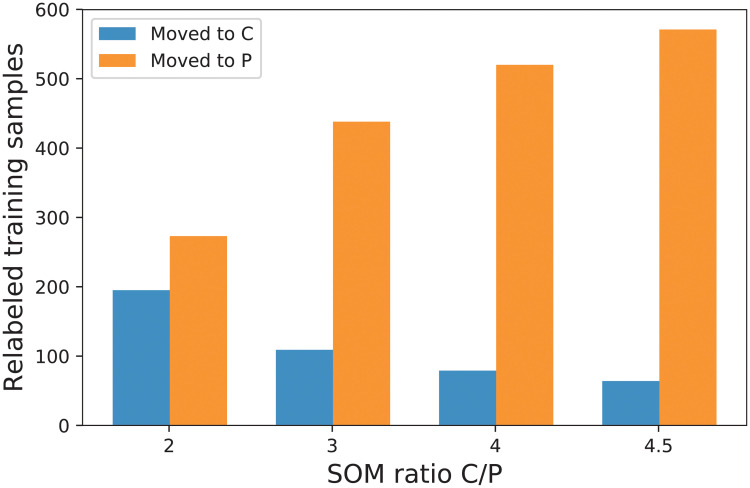
The number of training samples that are relabeled from C to P and vice versa when varying the ratio between C and P as found in the SOM map. In all cases, there are only 22 saccades that are relabeled as S from P.

Considering the poor results of the direct application of the DL algorithm to the original data, we decided that, due to the nature of the presymptomatic class, an unsupervised methodology would be beneficial in separating the saccades of the different classes and such a delineation could be useful for *cleaning* the training data set. Consequently, a second experiment, which is the one described in Algorithm 1, was performed by applying the SOM on the training data. Values for the *threshold* parameter are tried from the set {2, 3, 4, 4.5} in search for a better accuracy for the presymptomatic class. For example, a value of 2 means that the number of items labeled as C (control class) in a location of the SOM matrix has to be at least double the number of samples labeled as P (presymptomatic group) in order to assign the control class to saccades corresponding to that position. Fig 6 shows the number of training saccades that change their class to control or to presymptomatic depending on the established ratio threshold. Naturally, the higher the ratio threshold, the more saccades get labeled as presymptomatic and the less as control. In order to have a reference with respect to the number of relabeled samples, we mention that the total number of training saccades is 2245. The illustrated parameter obviously does not affect the number of saccades relabeled as sick, which is 22 in all cases.

**Fig 7:**
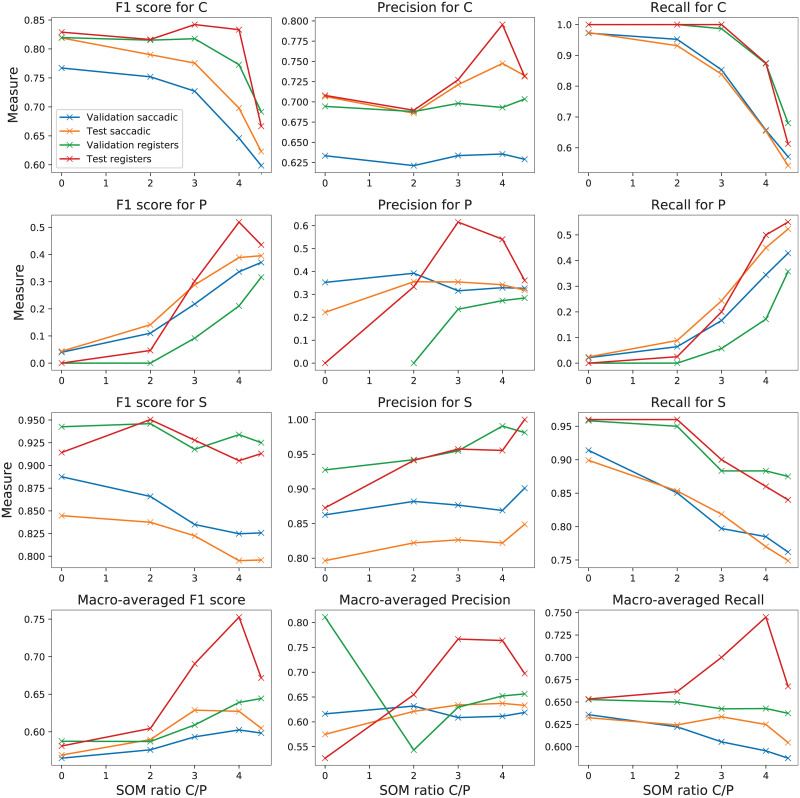
F1 score, precision, recall for C (first row), P (second row), S (last row) and macro-averaged over all classes respectively, for validation and test sets and for saccades and registers, with the variation of the *threshold* parameter (from training) on the horizontal axis. The 0 corresponds to the case where no relabeling is done. The legend from the first plot is considered for all plots.

Fig 7 outlines the F1 score, precision and recall for each of the three classes in turn and for each tried threshold. The SOM ratio C/P with a null value corresponds to the case where the original training was kept. The four representations correspond to the validation result on saccades and registers, as well as the corresponding test result for the same saccades and registers. As mentioned before, all results are calculated as a mean from 10 repeated runs. As it can be observed, the recall (sensitivity) of C and S are very well distinguished for ratio zero. This behaviour replicates the one noticed from Fig 5 regarding the control and sick classes when the classification is made on the original data. Similarly, the results for the F1 score are very good for these classes when the same ratio threshold is null. The precision also takes into account the number of samples that are mistaken from the specific classes. Since our goal was to increase the sensitivity for presymptomatic, the focus will especially stay on the recall results when choosing the most appropriate value for the threshold. It can be noticed that the higher the value for the ratio, the better are the recall results for class P. However, this happens at the cost of decreasing the sensitivity of the other two classes, so a convenient solution from the validation set needs to be selected. Since we are eventually interested in the accuracy per registers, the appropriate ratio is selected especially based on the green line (corresponding to validation on registers) from the recall plots. A good compromise is considered for a ratio threshold of 4. For a higher ratio value (i.e., 4.5), although the recall result is considerably better for class P, the decrease for class C is too high to accept this parameter value. This takes a descending trend from 4 to 4.5 in the SOM ratio threshold also in the last plot from the bottom row that illustrates the macro-averaged values. The same trend is verified for the F1 score for these two classes.

Fine tuning the SOM ratio parameter was also considered for values between 3 and 4, but these surprisingly led to similar relabeling as the case of 4. The precision is not highly influenced by the changes in the ratio for C and S, while the results improve for P when increasing the threshold. In average over all classes, there is a slight improvement, as seen in the middle plot from the bottom row in Fig 7. Since the F1 score is based on both precision and recall, it shows a more refined trend than recall. Naturally, the same threshold of 4 represents the adequate compromise between the gain in class P and the loss in class C for the F1 score.

**Fig 8:**
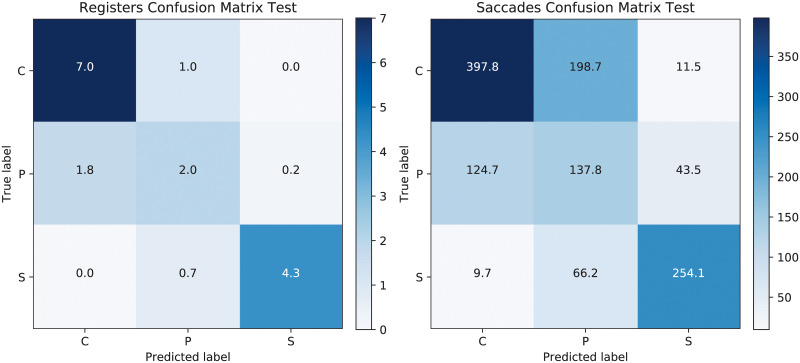
Confusion matrices for the test set after SOM-CNN-LSTM with ratio 4, using registers on the left and saccades on the right. The test prediction accuracy at the register level is 78.24%.

Fig 8 illustrates the confusion matrices for the test set for both registers and saccades when the SOM ratio C/P was taken as 4. Half of the P test registers are correctly classified at the cost of mistaking one register from the C to P and 0.7 (as averaged over 10 repeated runs) from S to P. Interestingly, the confusion matrix for the saccades indicate that although one third of the C samples are mistaken as P, there is only one register mistaken for this class. The classification test accuracy is relatively similar to the one obtained for the situation where the original data set was used, but this time there are also two registers from P correctly identified. The test result for the registers is of 78.24%, while the one for saccades is of 63.47%. The corresponding results for validation are of 73.23% and 60.82%, respectively. Alternatively, a single CNN architecture was tried for the same problem, as before in [4], but its results were significantly worse than those of the CNN-LSTM approach.

## 6 Conclusions

A SOM model is proposed in the current study with the aim of cleaning the training data. The necessity for using an unsupervised approach comes from the particularity of the problem that was being dealt with. The saccades are organized in registers and the class of each register establishes the class of the saccades it contains. However, the presymptomatic registers contain both healthy saccades, and some presymptomatic samples that are more similar to the sick ones. When using the saccades with the actual classes as given by their registers, the CNN-LSTM model is misled in the mission of delineating between healthy, presymptomatic and sick samples. An unsupervised approach like SOM finds characteristics from each class in turn and forms a map in which the neighbors exhibit similarities. Then, since the data is also unbalanced, in order to consider a position on the SOM map as representative for the control class, the score for this class needs to be four times higher than the one for presymptomatic. This threshold does not only take care of the unbalanced data, but also represents a further assurance that the regions on the SOM map indeed correspond to control saccades since so many training samples from class healthy were assigned to those positions.

An adequate threshold for the ratio between control and presymptomatic is selected based on the recall of the healthy and presymptomatic results for the validation registers. Using the relabeling by SOM and the C/P ratio of 4, the test accuracy for the registers was of 78.24% and half of the presymptomatic registers were correctly classified.

Further improvement in the classification accuracy may be achieved on the one hand by further fine tuning the hyper-parameters of the deep learning model, e.g. by means of a heuristic [14, 35], or, on the other hand through the use of additional information, like age, stimulation angle or saccade amplitude, beside the saccadic shape. Also a higher variety of DL model architectures will be tried in the future work, as others may lead to improved results.
